# Characterization of 29 polymorphic microsatellite markers developed by genomic screening of Sumatran rhinoceros (*Dicerorhinus sumatrensis*)

**DOI:** 10.1186/s13104-021-05522-x

**Published:** 2021-03-26

**Authors:** Jessica R. Brandt, Sinta H. Saidah, Kai Zhao, Yasuko Ishida, Isabella Apriyana, Oliver A. Ryder, Widodo Ramono, Herawati Sudoyo, Helena Suryadi, Peter J. Van Coeverden de Groot, Alfred L. Roca

**Affiliations:** 1 Department of Animal Sciences, University of Illinois Urbana-Champaign, Urbana, IL 61801 USA; 2 Department of Biology, Marian University, Fond du Lac, WI 54935 USA; 3 Genome Diversity and Diseases Laboratory, Eijkman Institute for Molecular Biology, Jl. Diponegoro No. 69, Jakarta, 10430 Indonesia; 4 Institute of Conservation Research, San Diego Zoo Global, Escondido, CA 92027 USA; 5 Rhino Foundation of Indonesia, Jl. Bima IV/10, Bogor, 16153 Indonesia; 6 Department of Biology, Queen’s University, Kingston, ON K7L 3N6 Canada; 7 Carl R. Woese Institute for Genomic Biology, University of Illinois Urbana-Champaign, Urbana, IL 61801 USA

**Keywords:** *Dicerorhinus sumatrensis*, Short tandem repeats, Non-invasive sampling

## Abstract

**Objective:**

The Sumatran rhinoceros is critically endangered, with fewer than 100 individuals surviving across its current range. Accurate census estimates of the remaining populations are essential for development and implementation of conservation plans. In order to enable molecular censusing, we here develop microsatellite markers with amplicon sizes of short length, appropriate for non-invasive fecal sampling.

**Results:**

Due to limited sample quantity and potential lack of genome-wide diversity, Illumina sequence reads were generated from two Sumatran rhinoceros samples. Genomic screening identified reads with short tandem repeats and loci that were polymorphic within the dataset. Twenty-nine novel polymorphic microsatellite markers were characterized (*A* = 2.4; *H*_*O*_ = 0.30). These were sufficient to distinguish among individuals (*P*_*ID*_ < 0.0001), and to distinguish among siblings (*P*_*ID(sib*)_ < 0.0001). Among rhinos in Indonesia, almost all markers were established as polymorphic and effective for genotyping DNA from fecal samples. Notably, the markers amplified and displayed microsatellite polymorphisms using DNA extracted from 11 fecal samples collected non-invasively from wild Sumatran rhinoceros. These microsatellite markers provide an important resource for a census and genetic studies of wild Sumatran rhinos.

**Supplementary Information:**

The online version contains supplementary material available at 10.1186/s13104-021-05522-x.

## Introduction

In the past two decades the population of Sumatran rhinoceros (*Dicerorhinus sumatrensis*) has declined by more than 50% [1]; with less than one percent of its former geographic range occupied and < 100 individuals of this critically endangered species surviving in isolated populations in Sumatra and Borneo. Despite the need for an accurate census and determination of the remaining genetic diversity for the conservation and management of surviving populations, an accurate census of the remaining populations has been elusive.

Molecular tools to amplify DNA from dung would be of great utility for a critical census of Sumatran rhinoceros. With rigorous fecal collection protocols followed, DNA from dung samples has been used to estimate population sizes in wildlife [2, 3]. Microsatellite markers are ideal for genetic profiling of populations due to high rates of evolution leading to intra-species polymorphisms, well-understood mutation dynamics, and proven utility in wildlife management and conservation of non-model taxa [4, 5]. Dung sample collection is relatively straightforward, may allow for a large proportion of the population to be sampled, and involves no stress from handling or direct observation of individuals [6]. Further, dung sampling allows for real time monitoring of changes in genetic diversity and population dynamics [7].

The evaluation of microsatellite DNA profiles from dung samples poses two challenges. First, dung samples contain a low quantity and quality of host DNA, as well as inhibitors and contaminants [8–10]. Both of these lead to reduced microsatellite amplification success with DNA from dung when compared to DNA from blood or tissue samples. Second, there are a limited number of Sumatran rhinoceros microsatellite markers, and these were not optimized for use with dung DNA. More specifically, the amplicon length had not been minimized [11].

## Main text

To generate more precise and accurate census estimates from in situ Sumatran rhinoceros populations, here we report optimized new microsatellite markers that target short regions of Sumatran rhinoceros DNA. We provide evidence of the amplification reliability and the presence of polymorphisms in Sumatran rhinoceros genotypes from dung. Specifically, we report the (a) identification of 29 novel short amplicon polymorphic Sumatran rhinoceros microsatellite markers, (b) characterization of variability of these markers using high quality DNA, (c) optimization of amplification success and verification of polymorphisms of these markers using DNA from dung, and (d) first field results from use of these markers on 11 dung samples from free-ranging Sumatran rhinoceros in Indonesia. Our results suggest that these new tools will be of value in the conservation and management of extant Sumatran rhinoceros.

### Methods and materials

#### Sample collection and DNA extraction

We used several types of Sumatran rhino samples (Table 1): previously extracted high quality DNA (*n* = 6) from the San Diego Zoo Institute for Conservation Research (ICR) and the Royal Ontario Museum (ROM); blood samples from rhinos kept at the Cincinnati Zoo (*n* = 2) or at the Sumatran Rhino Sanctuary (SRS) within Way Kambas National Park (WK) in Sumatra (*n* = 3); fecal samples from captive rhinoceros at the Cincinnati Zoo (*n* = 2) and SRS (*n* = 3); and fecal samples (*n* = 11) from an unknown number of wild rhino individuals at the Burkit Barisan Selatan National Park (BBS) in Sumatra. Across these samples, there were paired blood-fecal samples (each pair from the same individual) for three of the rhinos (two at the Cincinnati Zoo and one at SRS).

**Table 1:** Sumatran rhinoceros sample information

Sample	Specimen Type	Name	Source	Sex	Acquisition	Studbook Number	Geographic Origin
	*Samples tested at the University of Illinois*						
Dsu-33	DNA	Rami	San Diego Zoo (ICR)	F	1991	33	Sumatra
Dsu-35	DNA	Tanjung	San Diego Zoo (ICR)	M	1980	35	Sumatra
Dsu-28	Blood/fecal	Ipuh	Cincinnati Zoo	M	1980	28	Sumatra
Dsu-29	DNA	Emi	Royal Ontario Museum	F	1988	29	Sumatra
Dsu-63	DNA	Merah	Royal Ontario Museum	F	1980	19	Malay Peninsula
Dsu-64	DNA	Minah	Royal Ontario Museum	F	1987	15	Malay Peninsula
Dsu-66	DNA	Panjang	Royal Ontario Museum	F	1983	13	Malay Peninsula
Dsu-44	Blood/fecal	Harapan	Cincinnati Zoo	M	2007^a^	44	Captive born
	*Samples tested at the Eijkman Institute for Molecular Biology*						
Andalas-B	Blood	Andalas	Sumatran Rhino Sanctuary	M	2001^a^	42	Captive born
Andalas-D	Fecal	Andalas	Sumatran Rhino Sanctuary	M	2001^a^	42	Captive born
Rosa	Blood	Rosa	Sumatran Rhino Sanctuary	F	2005	45	BBS, Sumatra
Bina	Blood	Bina	Sumatran Rhino Sanctuary	F	1998	32	Bengkulu, Sumatra
Ratu	Fecal	Ratu	Sumatran Rhino Sanctuary	F	2005	46	WK, Sumatra
Andatu	Fecal	Andatu	Sumatran Rhino Sanctuary	M	2012^a^	50	Captive born
BBS1-1	Fecal	BBS-1–001	Free-ranging	F		NA	BBS, Sumatra
BBS1-15	Fecal	BBS-1–015	Free-ranging	F		NA	BBS, Sumatra
BBS1-17	Fecal	BBS-1–017	Free-ranging	F		NA	BBS, Sumatra
BBS3-5	Fecal	BBS-3–005	Free-ranging	M		NA	BBS, Sumatra
BBS3-6	Fecal	BBS-3–006	Free-ranging	F		NA	BBS, Sumatra
BBS3-10	Fecal	BBS-3–010	Free-ranging	F		NA	BBS, Sumatra
BBS3-13	Fecal	BBS-3–013	Free-ranging	F		NA	BBS, Sumatra
BBS3-17	Fecal	BBS-3–017	Free-ranging	F		NA	BBS, Sumatra
BBS3-19	Fecal	BBS-3–019	Free-ranging	M		NA	BBS, Sumatra
BBS3-25	Fecal	BBS-3–025	Free-ranging	F		NA	BBS, Sumatra
BBS3-29	Fecal	BBS-3–029	Free-ranging	F		NA	BBS, Sumatra

Whole genome sequences were generated from Dsu-33 and Dsu-35

^a^Indicates the year of birth. ICR is the Institute for Conservation Research. BBS is Burkit Barisan Selatan National Park. WK is Way Kambas National Park. NA is not applicable. Acquisition dates for the Sumatran Rhino Sanctuary (SRS) may be dates acquired from the wild or from another institution. Andalas was born in the Cincinnati Zoo in 2001 to Emi and Ipuh; Andatu is son of Andalas and Ratu. Bina was brought to the Safari Zoo in 1991 and moved to SRS in 1998. Sex of free-ranging rhinos was determined using molecular sexing (unpublished)

At the University of Illinois at Urbana-Champaign (UIUC), high quality DNA was extracted from the Cincinnati Zoo blood samples (*n* = 2) using the Qiagen DNeasy Blood and Tissue Kit. At the Eijkman Institute for Molecular Biology (EIMB), DNA was extracted from the SRS rhino blood samples (*n* = 3) using a salting out procedure [12]. At both UIUC and EIMB, fecal DNA was extracted from Cincinnati Zoo (*n* = 2), SRS (*n* = 3) and BBS (*n* = 11) using the QIAmp DNA Stool Kit (Qiagen) with a modified protocol [13].

#### Bioinformatic identification of polymorphic microsatellite loci

Whole genome sequences (Illumina MiSeq v3) were generated from two high quality DNA samples from Sumatran rhinoceros Dsu-33 and Dsu-35, both originally from Sumatra (Table 1). Reads from both rhinos were combined, and reads with short tandem repeat (STR) sequences that corresponded to the same locus were identified. Only those combined reads that exhibited polymorphism at an STR locus (across the four chromosomes sequenced) were used to design primers, using MsatCommander v1.0.8 [14]. To increase PCR success with the degraded Sumatran rhino DNA available from dung, primers were designed to amplify a short target product with a maximum length of 150 bp (inclusive of the primer lengths which total 36 to 44 bp). After running in silico* PCR* with the IPCRESS program [15], loci showing repetitive elements, monomorphism, a very broad allele size range, or sequences closely matching human were all excluded from consideration.

#### PCR amplification and locus polymorphism

Candidate primer pairs were evaluated at UIUC (Additional file 1) for amplification reliability and accuracy using available high quality (non-fecal) DNA from six Sumatran rhinos that had not been used in marker development, from the ICR and ROM (Table 1). A standard PCR mix and amplification protocol (Additional file 1) were used to test each of the candidate primer pairs. An agarose gel was used to verify a single amplicon of the correct size.

#### Estimates of microsatellite variability

Variability at the loci was characterized using six high quality DNA extracts (rhinos Dsu-28, 29, 44, 63, 64 and 66; Table 1). FSTAT, v2.9.3.2 [16] and GenAlEx, v6.1 [17, 18] were used to calculate the number of alleles per locus (*A*), expected heterozygosity (*H*_*E*_), and observed heterozygosity (*H*_*O*_). CERVUS v3.0.7 [19] was used to calculate the probabilities of individual identity, i.e., *P*_*ID*_ and *P*_*ID(sib)*_ values (for unrelated individuals and full siblings, respectively) for each marker, and cumulative *P*_*ID*_ and *P*_*ID(sib)*_ across multiple markers, using the equations of Waits et al. 2001 [20]. Cumulative *P*_*ID*_ and *P*_*ID(sib)*_ were calculated by multiplying *P*_*ID*_ and *P*_*ID(sib)*_ across loci. Using GENEPOP (https://genepop.curtin.edu.au/), significant linkage disequilibrium was not detected among loci. Genome scaffolds were identified for loci (Additional file 1: Table S1).

#### Amplification and genotyping of DNA from fecal samples of rhinos in Indonesia

To ensure that reliable and accurate dung genotyping of wild Sumatran rhinoceros can be conducted in the range country of Sumatran rhinos, we completed similar evaluations of DNA extraction protocols and PCR optimization steps at UIUC and EIMB. At EIMB, the Qiagen Multiplex PCR kit proved useful for amplifying low-quality DNA from dung samples collected at SRS and BBS (Additional file 1: Figure S1), with the PCR results evaluated for each marker for: shape of peaks, secondary peaks, DNA slippage bands, intensity in relative fluorescent units, and ease of allele identification. These observations were considered in compiling a set of markers that is more highly recommended for use, listed first in Table 2.

**Table 2:** Characterization of 29 microsatellite markers in Sumatran rhinoceros

Locus	Primer Sequence (5′–3′)	*A*	Size Range (bp)	*H*_*E*_	*H*_*O*_	*F*_*IS*_	*P*_*ID*_	*P*_*ID(sib)*_
	*Highly recommended*							
*Disu033*	F: TCTGGATACCTGAGGCTTGAC	2	152–164	0.53	0.00	1.00	0.41	0.63
	R: ACTGGCATCACTTCTTTCCC							
*Disu098*	F: GCTAGGAGAGGGTGTTGGAC	4	98–126	0.78	0.20	0.76	0.14	0.44
	R: TGGTAGCCTTGCCTCTTTCC							
Disu100*	F: TGTGGACTTGTCATATATGGGC	2	120–122	0.36	0.40	− 0.14	0.51	0.72
	R: TTCATCCATGCTGTCACAAATG							
*Disu201*^*‡*^	F: TGGAGAGAATTTCAGACATGGG	2	156–158	0.53	0.00	1.00	0.39	0.61
	R: CTAGCCCAAGATCCATTGGC							
*Disu261*	F: AAACCATACGCGGGAGAAGG	2	150–166	0.60	0.33	0.50	0.38	0.59
	R: GAAGGGAAGATCATGCAGGAG							
*Disu393*	F: AGTGAGCAAGGGAATGTGTG	2	155–157	0.36	0.40	− 0.14	0.51	0.72
	R: GGGTGCTGTCTCTTGATTGG							
Disu448*	F: CAGGTTTCGTTACTGCAGGAC	2	154–156	0.20	0.20	–	0.69	0.83
	R: TCTGGTGACCTGAGATGCAC							
Disu476*	F: AAACAGGGAAACAAGGTGCG	3	162–174	0.60	0.80	− 0.39	0.29	0.55
	R: GACTGCGCCCTTTCTGTTAG							
Disu487*^‡^	F: TATCATGTCACAAGCACGCG	2	148–160	0.20	0.20	–	0.69	0.83
	R: GTCTTCTTCACGACAGCACC							
Disu783*	F: CCTTGCCTTGCCTTCAATCC	3	126–134	0.51	0.60	− 0.20	0.34	0.61
	R: CCATCCTTTCTCCTACACAGAC							
*Disu847*	F: AAAGTCGCCTCTCACACACC	2	138–140	0.20	0.20	–	0.69	0.83
	R: TCAGAGCCTCCTTGTAAGCG							
	*Recommended*^*#*^							
*Disu050*	F: CTCCCACATTCAGCAAACTTTC	3	160–166	0.51	0.20	0.64	0.34	0.61
	R: CCAGGCAGTGATGACTCTAC							
*Disu071*^*‡*^ +	F: TTGAGATGCATTGCCGTGG	3	168–172	0.73	0.33	0.60	0.23	0.50
	R: CCATGGTTTCTGCATCGTGG							
*Disu076* +	F: TTCCAGCCGCTCTTATGACC	2	125–129	0.53	0.00	1.00	0.41	0.63
	R: TCATGTGCTTATTGGCCATCTG							
*Disu127*	F: CCACCACCACCATGCATAG	2	162–164	0.36	0.00	1.00	0.51	0.72
	R: CATTTGCTCCCATGCTGAAG							
*Disu138*	F: GGGACACATGACTCCTCTTATC	2	167–169	0.53	0.00	1.00	0.41	0.63
	R: CCACTCCACCTTATACTACCAC							
*Disu149*	F: GAGCGTGCATGGTAGTTTCC	4	160–168	0.73	1.00	− 0.43	0.18	0.47
	R: GGTTCTCATAGCAGACGGAG							
*Disu151*^*‡*^ +	F: CATTGTGCTCGCTACGCAG	2	135–137	0.36	0.00	1.00	0.51	0.72
	R: CTAGGTGTCAAGAGCCAGGG							
*Disu480*	F: CCTGCCTTCTAGTCCTGTGG	2	112–116	0.47	0.20	0.60	0.42	0.65
	R: AGCAAGCAGGATCAGGAAGG							
*Disu501*	F: TGGCCACATCTTCAGCATTAAG	2	155–157	0.47	0.60	− 0.33	0.42	0.65
	R: GCACCTAACACAGTTACAGGC							
*Disu542*^*‡*^ +	F: AAACTACAGGCACGTACAGC	2	128–130	0.20	0.20	–	0.69	0.83
	R: TTGAGAGATGAGGTGCGGTC							
*Disu545*	F: TGTTGTCCAAGCTGTGTCTG	2	148–150	0.20	0.20	–	0.69	0.83
	R: TGGCAGCTGGTACCTAACAG							
*Disu556* +	F: GCCAATTAAATCTACCTGCCAC	2	168–174	0.25	0.25	–	0.63	0.80
	R: GCCAAGACTCAAACCCAGG							
*Disu582*	F: TCTGTGGTGGTAGCTGTGAC	2	144–152	0.36	0.00	1.00	0.51	0.72
	R: TGGCACAGAGACACCCATG							
*Disu593*^*‡*^ +	F: CCACGTCCCAGGTCAAGAG	3	164–166	0.56	0.20	0.67	0.38	0.59
	R: AGCTGTTCCTGGTGGCTC							
*Disu733*	F: TGGCACAGAGACACCCATG	2	151–159	0.36	0.00	1.00	0.51	0.72
	R: TCTGTGGTGGTAGCTGTGAC							
*Disu748*^*‡*^	F: CCTTGATTGGTGGGTTCCC	3	106–116	0.64	0.80	− 0.28	0.26	0.53
	R: AGAGAGAGCGCACGTGTG							
	*Not recommended*							
*Disu269*^*‡*^ + *a*	F: CAAGACCACACCTGCTTGTC	3	115–152	0.60	0.33	0.50	0.30	0.58
	R: ACTCACTCATCACCCAGCC							
*Disu863*^*‡*^ + *b*	F: GAAGCTGTATGTCCGGATGC	2	162–166	0.36	0.40	− 0.14	0.51	0.72
	R: GCTAAACAGACCTTCCTCAGAG							

*A* is the number of alleles per locus, *H*_*E*_ and *H*_*O*_ are expected and observed heterozygosity, respectively. *P*_*ID*_ is the probability of identity and *P*_*ID(sib)*_ is the probability of identity between siblings. *F*_*IS*_ reflects deviation from Hardy–Weinberg proportions. Results are based on samples of six individuals initially tested. Among the highly recommended markers, an asterisk (*) indicates those that produced exceptionally good results. A dagger (^‡^) indicates that primers amplify tapir amplicons (though with very different sizes than for rhino); species identity may be established with mtDNA. A plus sign ( +) indicates primers that may amplify human DNA (see Table S4 for differences in size range). The primers listed as "recommended" (#) may be subject to further improvement with additional optimization. Reasons primers were not recommended: a-failed to amplify; b-lack polymorphism in Indonesian samples. The size range includes an M13 forward sequence (TGTAAAACGACGGCCAGT) added to the 5′ end of each forward primer (but not shown as part of the forward primer sequences above). The P_ID_ and P_ID(sib)_ are calculated by the method of Waits et al. 2001 (reference 20)

### Results

Our bioinformatics pipeline saved time and resources, because the candidate microsatellite loci chosen for further evaluation were polymorphic in two sequenced Sumatran rhinos. Of a total 30,556,224 Illumina sequencing reads, 176,357 reads (2.4%) from rhino Dsu-33 and 167,849 reads (2.8%) from Dsu-35 contained microsatellite motifs. Our bioinformatics method identified 861 polymorphic di-, tri-, and tetra-nucleotide repeat loci within the two sequenced Sumatran rhinos of which 229 had suitable priming regions. Loci were then excluded if they displayed < 6 tandem repeats, had too broad a size range, showed evidence of being in repetitive elements, or closely matched sequences of human DNA (a potential contaminating factor).

The above resulted in 55 polymorphic candidate loci for UIUC laboratory PCR optimization using six test samples (Dsu-28, 29, 44, 63, 64 and 66; Table 1). From the optimization efforts, 53/55 primer pairs produced amplicons within the expected size range when tested using at least two samples; while 24/53 were excluded for having secondary bands outside of the expected size range, for genotypes that were difficult to score, or for being monomorphic in the test samples. The monomorphic loci in the test samples suggest that some polymorphisms detected in the two Illumina-sequenced rhinos (different individuals from the six rhinos used to characterize variability) were due to low-frequency alleles. The polymorphism of the 29 remaining markers (Table 2; Additional file 1: Table S2) was characterized across the test rhinos: average allelic richness (*A*) = 2.4; number of alleles ranged from two to four; average expected heterozygosity (*H*_*E*_) = 0.45; and average observed heterozygosity (*H*_*O*_) = 0.30. Overall, *F*_*IS*_ = 0.44, likely due to population structure among geographically separated populations, or to inbreeding. Using the most informative markers, i.e., those with the lowest *P*_*ID*_ values, as few as seven loci could cumulatively distinguish individual identity (cumulative *P*_*ID*_ < 0.0001 [20]) (Table 2). Using a more conservative standard, *P*_*ID(sib)*_, as few as 19 optimized loci could confidently distinguish individual identity among siblings (cumulative *P*_*ID(sib)*_ < 0.0001 [20]; Table 2).

DNA from the dung of the two rhinos at the Cincinnati Zoo was used at UIUC to optimize PCR mix and cycling conditions (Additional file 1). At EIMB additional optimization was completed for the 29 markers (Additional file 1: Figure S1), with polymorphisms examined across three blood and three fecal samples from five SRS rhinos (for one SRS rhino, both types of sample were obtained). To conserve DNA resources, not all samples were used to test each marker. Yet 28/29 markers successfully amplified, and 27 of these were polymorphic in initial testing. For 13 markers randomly chosen among the 27, all 6 SRS samples were used to test marker quality and polymorphism. For some of these 13 markers, genotypes could be established for all of the SRS rhino samples (Additional file 1: Table S3). For 4/6 SRS samples, all 13 of the markers generated genotypes; for the other two (both fecal) samples, eight and twelve markers were successful (Additional file 1: Table S3). To examine the accuracy of genotypes from fecal samples, three paired blood-fecal samples were available (Table 1). While the number is low, comparing genotypes based on the two types of samples (Additional file 1) indicated that fecal samples yield accurate genotypes.

To further test their utility for a census using dung collected from wild Sumatran rhinos, the 13 randomly chosen new markers were tested on 11 fecal samples collected from an unknown number of wild rhinoceros in BBS (Table 1). Their identity as rhino samples was established by mtDNA sequencing because wild rhino and Asian tapir (*Tapirus indicus*) fecal samples are sometimes difficult to distinguish in the field. The specificity of primer pairs was tested using Asian tapir DNA and human DNA (a conceivable contaminant). One or both of these species amplified for 12/29 markers, although their amplicon sizes were almost always substantially different from those of rhino (Additional file 1: Table S4).

After species identity was established for 11 fecal samples from wild rhinos, the 13 randomly chosen markers were tested. For nine of the 13 markers, DNA from least nine of the 11 fecal samples amplified successfully and could be scored (Additional file 1: Table S3). For DNA from nine of the 11 fecal samples from wild Sumatran rhinos, at least 9 of the markers amplified successfully. This survey thus established proof of principle for the utility of the markers for fecal censusing of wild Sumatran rhinoceros.

### Discussion

Recent estimates suggest that fewer than 100 Sumatran rhinoceros individuals persist across Sumatra, with few in Borneo, although current census estimates have a large degree of uncertainty [21]. Since an accurate census could guide management decisions, it is crucial to have reliable methods for non-invasively estimating population size. Overall, we generated a panel of polymorphic microsatellite markers useful for genetically distinguishing among individuals. They are appropriate for fecal DNA given their specificity for rhinos and short amplicon lengths. As almost all markers successfully amplified DNA from fecal samples, they will be useful for informing conservation managers about the population size and genetic characteristics of wild Sumatran rhinoceros.

## Limitations

As additional fecal samples are collected from wild rhinoceros, PCR conditions and marker choice may be further optimized for some primer pairs (Table 2). Although initial results suggest that the genotyping error rate is low, further testing of these markers using a larger number of paired blood and fecal samples is required to better estimate genotyping error (i.e., allelic dropout and false alleles) [22].

## Supplementary Information

**Additional file 1.** Detailed methods, supplementary figures and tables.

## Data Availability

Raw sequencing reads were deposited in the Sequence Read Archive at the NCBI: accession number SRR12548982 for Dsu-33 (BioSample accessions: SAMN15944577); and SRR12548981 for Dsu-35 (BioSample accessions: SAMN15944578). The datasets used and/or analyzed by the current study are available from the corresponding author on reasonable request. Sequences for each locus are shown in Additional file 1: Figure S2.

## Abbreviations

BBS: Burkit Barisan Selatan National Park
EIMB: Eijkman Institute for Molecular Biology
ICR: Institute for Conservation Research (San Diego Zoo)
ROM: Royal Ontario Museum
SRS: Sumatran Rhino Sanctuary
UIUC: University of Illinois at Urbana-Champaign
WK: Way Kambas National Park
