# Supplementary material for: Characterization of 29 polymorphic microsatellite markers developed by genomic screening of Sumatran rhinoceros (Dicerorhinus sumatrensis)
Source: BMC Res Notes. 2021 Mar 26;14:119. doi: 10.1186/s13104-021-05522-x (PMC7995689; doi:10.1186/s13104-021-05522-x)
Supplement: Supplementary file 1 — Additional file 1. Detailed methods, supplementary figures and tables. [file 13104_2021_5522_MOESM1_ESM.pdf]

Characterization of 29 polymorphic microsatellite markers developed by genomic screening of Sumatran rhinoceros (*Dicerorhinus sumatrensis*).

Additional file 1

<u>Detailed Methods, University of Illinois at Urbana-Champaign (UIUC)</u>

Sample collection and DNA extraction at the University of Illinois

Whole blood samples from rhinos at the Cincinnati Zoo (Table 1) were collected during routine veterinary care, stored in EDTA tubes, and refrigerated until DNA isolation (< 1 week from time of collection). DNA was isolated using the Qiagen DNeasy Blood and Tissue Kit (QIAGEN) according to the manufacturer's recommended protocol. One fecal sample from the Cincinnati Zoo (Dsu-44; Table 1) was collected fresh by veterinary staff using a sterile instrument (wooden tongue depressor) to scrape approximately 2 mL from the exterior of each sample into a collection tube. The sample was stored briefly (less than three days) at 4°C until shipment to the University of Illinois at Urbana-Champaign (UIUC). The second fecal sample (Dsu-28; Table 1) was collected from the individual post mortem and frozen at -20°C until shipment. Upon arrival to UIUC, 10-12 mL of a 20% DMSO salt saturated solution was added to each collection tube; samples were subsequently stored at -20°C. DNA was isolated from each dung sample using published modifications [1] to the manufacturer's recommended protocol for the QIAmp DNA Stool Kit (QIAGEN), with additional modifications as described below.

Genomic sequencing and bioinformatics

DNA samples of two rhinoceros (Dsu-33 and Dsu-35; Table 1) were sent to the UIUC Biotechnology Center for library preparation, including the addition of a unique identifying

barcode and sample pooling, and DNA sequencing on the Illumina MiSeq v3 platform. Resulting paired-end reads from Illumina MiSeq v3 sequencing of Dsu-33 and Dsu-35 with overlapping sequence (within each individual) were merged using FLASH 1.2.8. A program was written in C to take large genome-scale, unassembled high-throughput sequences and return microsatellite-containing reads for subsequent analysis. In this program, relaxed criteria for extraction of reads containing microsatellite motifs were used, thus filtering uninformative reads out of the working databases. The program selected reads with di-, tri- and tetra-nucleotide motifs containing at least four repeats. Reads with the tandem repeats in the first or last 50 nucleotides were removed to allow sufficient flanking region for primer design. The full sequence read was required to be a minimum of 120 bp in length.

A total of 30,556,224 sequencing reads were obtained, with 16,813,030 reads from Dsu-33 (average length of 410 bp) and 13,743,194 reads from Dsu-35 (average length of 440 bp). After paired-end sequences were joined, databases were created of 7,399,098 reads for Dsu-33 and 5,993,320 reads for Dsu-35. A total of 176,357 reads (2.4%) from Dsu-33 and 167,849 reads (2.8%) from Dsu-35 were found to contain microsatellite motifs with four or more tandem repeats.

To search microsatellite containing reads for potential polymorphisms, a Python-based script was written to combine all reads containing microsatellites from both rhinos into one database and subsequently omit the microsatellite motif from each read, leaving a set of flank-pairs (i.e., a pair of flanks from the same original read, one from either side of the microsatellite motif). A MegaBLAST pair-wise analysis, requiring 99% sequence identity and an ungapped alignment, was completed to identify matching flank-pair sequences. The sequences of matching flank-pairs were aligned and those containing microsatellite motifs with a differing number of tandem repeats were retained. For each alignment, a stitched sequence maximizing the length of the combined flanks was used as the representative

sequence for the locus.

The representative sequences of potentially polymorphic loci were further analyzed in MSATCOMMANDER 1.0.8 [2]. Sequences were again screened for di-, tri-, and tetra-, microsatellite motifs, this time with a minimum of six tandem repeats. Primers were designed in MSATCOMMANDER through an interface with PRIMER3 software [3] to meet the following criteria: amplification of a target product in the 75 to 150 bp size range (inclusive of the two primer lengths), optimal primer length of 20 base pair (range 18 to 22 base pair), optimal melting temperature of 60.0°C (range of 58.0°C to 62.0°C), optimal GC content of 50%, inclusion of at least 1 bp GC clamp, low self or pair complementarity and a maximum end stability of 8.0 [2].

Once the set of potential loci was identified, a number of quality checks and screening criteria were implemented before selection of primer pairs for testing in the laboratory. To determine if the designed primer pairs would produce amplicons of varying size (as expected at a polymorphic locus), the IPCRESS program [4] was used to run *in silico PCR*. Each primer pair was computationally "amplified" against the joined paired-end sequencing databases from Dsu-33 and Dsu-35. IPCRESS identified "amplicons" that would potentially be produced from each individual during PCR with no priming mismatches, one priming mismatch, and two priming mismatches. Primer sets that were expected to produce amplicons of a single size, or of more than four varying lengths in the *in silico* PCR step were not further considered, to avoid monomorphic loci or repetitive elements, respectively. Additionally, loci that exhibited broad size ranges (more than 20 bp difference between alleles) were excluded, to preclude potential non-specific amplification or amplification of loci containing indels or many null alleles. Remaining primer sequences and full amplicon sequences were searched against the non-redundant BLAST database (https://blast.ncbi.nlm.nih.gov/Blast.cgi). Any locus showing evidence of being part of a

repetitive element (e.g., LINEs or SINEs), or that closely matched sequences of human DNA (a conceivable contaminating factor) were screened out. After screening the reads containing microsatellite motifs, 861 potentially usable polymorphic loci were identified. Suitable priming regions were present for 229 of the loci; after the quality checks noted above were conducted, a set of 55 potentially polymorphic loci was identified as suitable for laboratory testing.

PCR and genotyping of high quality (i.e., non-fecal) DNA

All forward primers had an M13 forward (M13F) sequence (TGTAAAACGACGGCCAGT) added to the 5' end to allow fluorescent labeling as previously described (Schuelke 2000; Boutin-Ganache et al. 2001). A primer mix consisting of 8.5 μM reverse primer, 0.6 μM of M13F-tailed forward primer, and 8.5 μM of fluorescently labeled M13F primer was used for PCR. Primer pairs were initially tested by PCR performed in a 10 μL reaction mixture that included: 2 mM $MgCl_2$, 200 μM of each dNTP (Applied Biosystems Inc. [ABI]), 1x PCR buffer, and 0.4 units/μl final concentration of AmpliTaq Gold DNA Polymerase (ABI). Negative controls (water instead of DNA) were included with each PCR amplification. A "touchdown" PCR algorithm was used with an initial 95°C for 10 min; cycles of 15 sec at 95°C; followed by 30 sec at 60°C, 58°C, 56°C, 54°C, 52°C (2 cycles at each temperature) or 50°C (last 30 cycles); and 45 sec at 72°C; and a final extension of 30 min at 72°C. The amplicons were visualized on a 1.5%–2% agarose gel with ethidium bromide under ultraviolet light. PCR amplicons were diluted depending on the intensity of the signal on the agarose gel photograph then submitted to the University of Illinois at Urbana-Champaign High-Throughput Sequencing and Genotyping Unit to run on the ABI 3730XL capillary sequencer for fragment analysis. The software Genemapper version 3.7 (Life Technologies) was used to examine the microsatellite signals for secondary bands far outside the expected size range, and for low quality of results during fragment

analysis. Genotyping results were examined across six rhinos for polymorphisms. After the PCR results were thus analyzed, 29 of 55 primer pairs were retained for use.

For each of the 29 loci, sequences were queried using NCBI nucleotide blast (https://blast.ncbi.nlm.nih.gov/Blast.cgi?PROGRAM=blastn&PAGE_TYPE=BlastSearch&LINK_LOC=blasthome). Each locus sequence (Figure S2) was used as query for blastn, selecting the "Whole-genome shotgun contigs" database, with "Rhinocerotidae (taxid:9803)" selected as "organism." In Table S1, the highest scoring matches to genome scaffolds are listed for two subspecies of the Sumatran rhinoceros (*Dicerorhinus sumatransis sumatransis* and *D. s. harrissoni*) for which genome sequences are available (one individual of each subspecies).

Fecal DNA Extraction and Genotyping at the University of Illinois

Fecal samples were provided by the Cincinnati Zoo from the two Sumatran rhinoceros in North America at the time of this study; these samples were used for preliminary evaluation of genotyping success at UIUC. At UIUC, a number of modifications were tested to improve both fecal DNA extraction protocols and PCR amplification results.

The QIAmp DNA Stool Kit protocol [1] was used, with the following modifications initially tested at UIUC: each fecal sample was thoroughly homogenized in 20% DMSO salt saturated buffer by vortexing for 5 minutes; the initial sample volume was increased to 800 µl; samples were digested overnight in 1 mL of ASL buffer and 1 mg of proteinase K at 56°C; vortex times throughout were increased (especially for the InhibitEx step which was vortexed for 5 minutes); and final elution was done twice with 50 µl of elution buffer each time and a minimum 30 minute incubation at room temperature. DNA concentrations were measured using the Qubit 2.0 Fluorometer for the modified extraction protocol; resulting values were compared to those obtained with the standard protocol. Subsequent PCR results

suggested that these modifications to the extraction methods increased the amplification success, and subsequent optimization steps used DNA isolated following the modified protocol.

Various PCR conditions were also tested, and those that appeared to be more effective at improving amplification success were adopted. These tests included additional treatments to remove PCR inhibitors, various dilutions of the DNA extract, use of different DNA polymerases, variations in concentration of $MgCl_2$, varying the annealing temperature range of touchdown PCRs, the number of cycles at each temperature, and the lengths of the elongation step, and the amount of DNA extract used. With the caveat that the number of Sumatran rhino fecal samples was too limited to report results quantitatively, the following UIUC protocol incorporates the factors that appeared to improve success.

Genotyping was carried out using the following PCR conditions with the same primer mix mentioned above (in the section on PCR of higher quality DNA): 10 or 20 µl reaction mixture that included: 4 mM $MgCl_2$, 200 µM of each dNTP (Applied Biosystems Inc. [ABI]), 1µg/ul of bovine serum albumin (BSA; New England BioLabs Inc), 1X PCR Buffer II (ABI), 1.0 unit final concentration of AmpliTaq Gold DNA Polymerase (ABI), and 1 or 2 µl of template DNA. Negative (water) and positive (blood DNA) controls were included with each PCR set. A touchdown PCR algorithm was used with an initial denaturation at 95°C for 10 min; cycles of 15 sec at 95°C, followed by 15 sec at 66°C, 64°C, 62°C, 60°C, 58°C (4 cycles at each temperature) or 56°C (last 25 cycles), and 10 sec at 72°C; and a final extension of 30 min at 72°C. Amplification success was checked before submission to run on a capillary electrophoresis on the ABI 3730XL genetic analyzer at the UIUC Biotechnology Center as mentioned above. Fragments were assessed to determine if the markers produced readable peaks and if they were polymorphic in size using GeneMapper Version 3.7 software. Genotype data from fecal samples were compared to those generated using the higher quality

DNA from blood; instances of allelic dropout and false allele rate, which produce incorrect genotypes, were recorded.

Because the markers were intended for use in populations of Sumatran rhinoceros in their home range, additional tests of the markers were conducted in Indonesia at the Eijkman Institute for Molecular Biology.

Detailed Methods at the Eijkman Institute for Molecular Biology (EIMB)

Overview of PCR modifications

In Indonesia, additional troubleshooting and modifications were conducted, to account for the use of different platforms, and given that plants eaten by rhinoceros (which may contain inhibitors to PCR) would be different from those eaten by rhinoceros in North American zoos. Protocols were tested at the Eijkman Institute for Molecular Biology, for use with DNA from blood and fecal samples from the Sumatran Rhino Sanctuary (SRS), Way Kambas National Park, Indonesia and fecal samples from Burkit Barisan Selatan National Park (BBS), Indonesia. One modification was testing the use of reagents from the Qiagen Mutiplex PCR Kit. The PCR cycling used for the multiplex kit followed the manufacturer's recommendation and was different from the touchdown PCR algorithm applied when using AmpliTaq Gold Polymerase. The changes are incorporated into the protocol shown below. For two of the markers (*Disu100* and *Disu593*), PCR on the same samples was conducted separately using either AmpliTaq Gold DNA Polymerase or using the Qiagen Multiplex PCR Kit, with results for each shown in Figure S1, below. For both markers, using the Qiagen Multiplex PCR Kit yielded a better or stronger signal than the AmpliTaq Gold polymerase (Figure S1, below). Results for other markers are available upon request.

Sample Collection and DNA Extraction

Blood samples of captive individuals at SRS were collected during regular veterinary care. Whole blood was stored in EDTA tubes and refrigerated until DNA extraction (ca. 1 month). DNA was extracted using a salting out procedure [5]: Non-nucleated cell contamination was minimized by washing with red blood cell lysis solution three times. Samples were then incubated in cell lysis solution and RNase A for 2-4 hours. Ammonium acetate (5M) was added for protein precipitation. The subsequent step was DNA precipitation using isopropanol. Lastly, DNA samples were eluted in 100µl TE buffer and incubated for at least 2 hours. While the procedures used for DNA extraction varied between the laboratories (UIUC and Eijkman Institute) this would not have affected the utility of the DNA for PCR.

The fecal samples from captive individuals at the SRS were collected fresh. For fecal samples from wild individuals at BBS, more time may have elapsed before collection of the fecal samples (perhaps 24 hours - 2 weeks). The samples were taken using a wooden tongue depressor as described above, and were preserved in 20 mL 20% DMSO salt saturated buffer. After being transferred to the Eijkman Institute, fecal samples were stored at -20°C. DNA from fecal samples was isolated [1] using the QIAmp DNA Stool Kit (QIAGEN) with the same modifications as in UIUC (above). All DNA samples were then stored at -20°C for subsequent use.

DNA amplification and genotyping

PCR amplification was performed in a DNA-free laminar hood to prevent contamination. PCR that used Amplitaq Gold Polymerase followed the UIUC procedure described above, but this procedure performed poorly. Thus, the Qiagen® Multiplex PCR kit was employed, which generated improved results for genotyping. PCR products were fluorescently labeled using M13 forward (M13F)-tailed forward primer and the same primer mix as stated above for UIUC. The sample was amplified in 10 µL reaction consisting of PCR-grade water, 1x Master Mix, Q-Solution, primer mix and 100-200 ng DNA template for

fecal samples or 5 ng DNA template for blood samples. The difference in the amount of DNA used for dung and blood samples was intended to adjust the endogenous DNA amount and also to yield similar heights of peaks in genotyping results, because peaks from the blood sample DNA would tend to be higher. Amplification was carried out in the GeneAmp® CR System 9700 thermocycler (Perkins Elmer/ Applied Biosystems [ABI], Singapore), using the following cycling: 95°C for 15 min followed by 35 cycles of denaturation at 94°C for 30 sec, annealing at 57°C for 90 sec, extension at 72°C for 60 sec, and a final extension step at 60°C for 30 min. A positive control (DNA from a blood sample that had previously demonstrated amplification success) and negative (water) control were included with each run. After confirming the amplification with an agarose gel electrophoresis, capillary electrophoresis was performed using an Applied Biosystem® 3130 Genetic Analyzer (ABI, Foster City, CA, USA), each run containing a mix of: 0.2 µL of GeneScan™ 500 LIZ® molecular size marker, 9.3 µL of HiDi Formamide and 1 µL of PCR product (ABI, Foster City, CA). The results were scored using GeneMapper®V.4.0 software (ABI, Foster City, CA, USA).

Primer testing using human or tapir DNA

Tests using human and using tapir DNA were conducted for each primer pair using the Qiagen Multiplex PCR Kit. These were conducted because it is difficult to distinguish fecal samples from rhinoceros and tapir (*Tapirus indicus*) in the field. Also, human DNA may pose a risk of contamination during sampling or extraction. All the primer pairs were tested using DNA from one human and from one tapir sample. The methods used for this assessment are the same as for the singleplex rhinoceros PCR protocol mentioned immediately above.

<u>Testing of paired blood-fecal samples at EIMB and UIUC</u>

While not required for a preliminary examination of amplification success as a pilot

study, the genotyping of DNA from dung samples for a census of rhinos would benefit if each sample for each marker was genotyped multiple times, e.g., as described by Okello and colleagues [1].

To evaluate the precision and accuracy of genotypes generated with the new markers on fecal samples, paired blood and fecal samples were available at EIMB for one SRS rhino (Andalas) and at UIUC for two Cincinnati Zoo rhinos (Dsu-28 and -44) (Table 1).

For Dsu-44 (a high-quality fecal sample, see above), 6 loci that were tested in 3 separate reactions each showed no evidence of allelic dropout or false alleles in the dung samples when compared to genotypes from the (higher quality) DNA from blood. For the other 23 markers tested only once, genotyping was successful at 22 loci. By contrast, there were limited rates of success for Dsu-28 (a degraded fecal sample, see above) during three replication tests of six loci (the same six loci tested 3 times for Dsu-44), with PCR successful for the fecal DNA in only eight of the 18 reactions, most likely because the sample was of poor quality.

At EIMB, the fecal and blood DNA samples from Andalas were included in the testing of 13 randomly chosen loci using the SRS samples. For these 13 markers, genotypes were completely consistent in the paired blood-fecal samples, with no allelic dropout or false alleles in the fecal genotypes.

While the number of available paired blood-fecal samples was low, the results suggested that accurate genotypes were obtained from the fecal samples.

Detailed protocol

A step-by-step PCR protocol using the Qiagen Multiplex PCR Kit is included below.

PCR protocol for Sumatran rhino microsatellite analysis (using Multiplex PCR Kit)

1. All the microsatellite primers were provided by UIUC. The primers were eluted into 100 µM home stock and 20 µM working stock.
2. Markers *Disu100* and *Disu733* were employed to test this protocol. The primer mix was prepared using the same procedure described above for UIUC.
3. This protocol is for singleplex PCR using the Qiagen® Multiplex PCR kit.
4. Mixing of PCR reagents (components are listed in step 6) and applying the cocktail to the reaction tubes or plates were conducted in a Biosafety Cabinet exclusively for PCR setup.
5. DNA template was added to the tubes or plates prepared in step 4 in a separate laminar hood used for DNA only
6. Singleplex PCR mix is listed here:

Component	Final Conc.	Volume (µL) / 1 reaction
PCR grade water	-	1,7*
2x Master Mix	1 x	5
Q Solution	-	2
Primer mix	-	0,3
Total volume to aliquot	-	9*
DNA template (100-200ng/µl)		1*

*adjust to total volume 10µL for each reaction, depending on volume of DNA template used.

7. After adding the DNA template, place in a Geneamp® PCR system 9700 with cycling conditions (hotstart) as follows:
   95°C for 15 minutes initial denaturation to activate HotStarTaq DNA Polymerase
   35 cycles of
   - 94°C for 30 sec denaturation
   - 55°C for 90 sec annealing
   - 72°C for 60 sec extension

   60°C for 30 minutes final extension
   Hold at 25°C (can also be held at 4°C)
8. Remove sample tubes immediately after the cycles are finished, store at -20°C until proceeding to capillary electrophoresis.
9. Capillary electrophoresis was conducted following the Genescan® protocol in an ABI 3130 Genetic Analyzer.
10. PCR product was mixed with Hi-Di™ Formamide and GeneScan™ -500 LIZ Size Standard as follows:

Component	Volume per 1 reaction
Hi-Di™ Formamide	9,3
GeneScan™ -500 LIZ Size Standard	0,2
PCR Product	1
Total	10,5

11. Finally, the result from electrophoresis visualization was interpreted, using GeneMapper v. 4.

References

1. Archie EA, Moss CJ, Alberts SC: **Characterization of tetranucleotide microsatellite loci in the African Savannah Elephant (*Loxodonta africana africana*)**. *Mol Ecol Notes* 2003, **3**(2):244-246.
2. Faircloth BC: **MSATCOMMANDER: detection of microsatellite repeat arrays and automated, locus-specific primer design**. *Molecular Ecology Resources* 2008, **8**(1):92-94.
3. Rozen S, Skaletsky H: **Primer3 on the WWW for general users and for biologist programmers**. *Methods in molecular biology (Clifton, NJ)* 2000, **132**:365-386.
4. Slater GS, Birney E: **Automated generation of heuristics for biological sequence comparison**. *Bmc Bioinformatics* 2005, **6**.
5. Miller SA, Dykes DD, Polesky HF: **A simple salting out procedure for extracting DNA from human nucleated cells**. *Nucleic Acids Res* 1988, **16**(3):1215.

Table S1: Genome scaffolds identified for microsatellite loci

query id	subject ids query acc.ver	% identity	alignment length	mismatches	gap opens	q. start	q. end	s. start	s. end	evalue	bit score
Disu033	JABWHU010001775.1	100	42	0	0	1	42	18960450	18960491	1.13E-11	77
Disu033	PEKH010002758.1	100	42	0	0	1	42	137033	137074	1.13E-11	77
Disu050	JABWHU010001767.1	76.667	150	33	1	1	150	1602193	1602046	4.15E-30	138
Disu050	PEKH010007781.1	79.333	150	31	0	1	150	5957	5808	1.27E-36	159
Disu071	JABWHU010000403.1	100	79	0	0	76	154	26884025	26883947	1.01E-31	143
Disu071	PEKH010005704.1	100	79	0	0	76	154	1601115	1601037	1.01E-31	143
Disu100	JABWHU010000554.1	100	32	0	0	1	32	41275668	41275699	2.02E-06	59
Disu100	PEKH010002612.1	96.875	32	1	0	1	32	1091494	1091463	8.60E-05	54.5
Disu127	JABWHU010001519.1	98.333	60	1	0	89	148	34637165	34637224	8.50E-20	104
Disu127	PEKH010002601.1	100	60	0	0	89	148	412605	412664	2.00E-21	109
Disu138	JABWHU010000011.1	100	54	0	0	44	97	6054918	6054865	3.30E-18	98.7
Disu138	PEKH010001193.1	100	97	0	0	1	97	547917	547821	1.50E-41	176
Disu149	JABWHU010001130.1	68.667	150	41	1	1	144	14076000	14075851	1.22E-17	97.8
Disu149	PEKH010007506.1	70.548	146	41	1	1	144	494355	494210	8.21E-20	104
Disu151	JABWHU010000040.1	100	42	0	0	79	120	81083718	81083759	8.96E-12	77
Disu151	PEKH010005509.1	100	42	0	0	79	120	1468073	1468032	8.96E-12	77
Disu201	JABWHU010001765.1	75.352	142	35	0	1	142	38708422	38708281	5.80E-28	131
Disu201	PEKH011061266.1	74.648	142	34	1	1	142	31430	31569	3.00E-25	122
Disu261	JABWHU010000040.1	74.194	155	34	1	1	149	1532461	1532615	2.15E-27	130
Disu261	PEKH010004475.1	77.852	149	23	1	1	149	1094482	1094344	2.80E-32	145
Disu393	JABWHU010000050.1	100	102	0	0	1	102	9741804	9741905	2.86E-44	185
Disu393	PEKH010006947.1	100	100	0	0	1	100	48778	48877	3.49E-43	181
Disu448	JABWHU010001519.1	100	25	0	0	115	139	9323827	9323851	0.018	46.4
Disu448	PEKH010001084.1	100	25	0	0	115	139	1067015	1066991	0.018	46.4
Disu476	JABWHU010001130.1	100	40	0	0	107	146	9738659	9738698	1.41E-10	73.4
Disu476	PEKH010004901.1	100	40	0	0	107	146	980919	980880	1.41E-10	73.4
Disu480	JABWHU010001777.1	100	40	0	0	63	102	66719392	66719353	8.68E-11	73.4
Disu480	PEKH010004887.1	100	40	0	0	63	102	637179	637218	8.68E-11	73.4
Disu487	JABWHU010000403.1	100	61	0	0	83	143	9946919	9946859	5.48E-22	111
Disu487	PEKH010002407.1	100	61	0	0	83	143	205461	205521	5.48E-22	111
Disu501	JABWHU010001769.1	100	44	0	0	94	137	43118096	43118053	8.78E-13	80.6
Disu501	PEKH010010134.1	100	44	0	0	94	137	241054	241011	8.78E-13	80.6
Disu542	JABWHU010000901.1	100	60	0	0	58	117	6558605	6558664	1.46E-21	109
Disu542	PEKH010001294.1	100	60	0	0	58	117	145113	145054	1.46E-21	109
Disu545	JABWHU010000397.1	100	49	0	0	86	134	779415	779463	1.65E-15	89.7
Disu545	PEKH010003208.1	100	49	0	0	86	134	149571	149523	1.65E-15	89.7
Disu556	JABWHU010000050.1	100	62	0	0	1	62	44710193	44710132	1.65E-22	113
Disu556	PEKH010004646.1	100	56	0	0	1	56	650651	650706	2.99E-19	102
Disu582	JABWHU010001771.1	100	46	0	0	86	131	11229140	11229095	6.79E-14	84.2
Disu582	PEKH010001376.1	100	46	0	0	86	131	265639	265594	6.79E-14	84.2
Disu593	JABWHU010001770.1	86.395	147	20	0	1	147	2789913	2790059	2.11E-46	193
Disu593	PEKH010002485.1	85.906	149	19	1	1	147	969701	969849	2.57E-45	189
Disu733	JABWHU010001771.1	100	46	0	0	1	46	11229095	11229140	6.79E-14	84.2
Disu733	PEKH010001376.1	100	46	0	0	1	46	265594	265639	6.79E-14	84.2
Disu748	JABWHU010001775.1	100	34	0	0	65	98	8689428	8689395	1.48E-07	62.6
Disu748	PEKH010003326.1	100	34	0	0	65	98	85975	86008	1.48E-07	62.6
Disu783	JABWHU010000747.1	100	47	0	0	1	47	18030046	18030000	1.55E-14	86
Disu783	PEKH010002757.1	100	47	0	0	1	47	1749798	1749752	1.55E-14	86
Disu783	PEKH010009812.1	100	47	0	0	1	47	6026	5980	1.55E-14	86
Disu847	JABWHU010000040.1	100	50	0	0	1	50	5622102	5622151	4.44E-16	91.5
Disu847	PEKH010000243.1	100	50	0	0	1	50	2147767	2147816	4.44E-16	91.5
Disu863	JABWHU010001519.1	100	53	0	0	96	148	58023207	58023155	1.26E-17	96.9
Disu863	PEKH010000692.1	100	53	0	0	96	148	3585016	3584964	1.26E-17	96.9

For each microsatellite locus sequence, the best scoring match is given for two subspecies of Sumatran rhinoceros: *Dicerorhinus sumatrensis sumatransis* (PEKH01) and *D. s. harrissoni* (JABWHU01). In no case did a locus show the large number of matches indicative of a repetitive element. The few not listed had short and repetitive sequences: *Disu076* did not have a Blastn match to the Sumatran rhinoceros, but did produce a match to the white rhinoceros, *Ceratotherium simum*.

Table S2. Testing of locus variability in high quality samples.

		Sumatran Rhinoceros Individuals											
		Dsu-28		Dsu-29*		Dsu-66		Dsu-64		Dsu-63*		Dsu-44*	
Microsatellite Loci	Disu542	128	130	128	128	128	128	128	128	128	128		
	Disu501	155	157	155	157	155	155	155	157	155	155		
	Disu556	174	174	168	174	174	174	174	174	000	000		
	Disu863	162	162	162	162	162	166	162	162	162	166		
	Disu448	154	156	156	156	156	156	156	156	156	156		
	Disu201	156	156	156	156	158	158	158	158	156	156		
	Disu847	138	138	138	138	138	138	138	140	138	138		
	Disu393	155	157	155	157	155	155	155	155	155	155		
	Disu733	151	151	151	151	151	151	159	159	151	151		
	Disu149	160	162	160	168	160	162	162	166	160	162		
	Disu783	126	128	126	128	126	126	126	126	126	134		
	Disu50	160	166	164	164	160	160	160	160	160	160		
	Disu748	110	116	106	116	106	116	106	116	106	106		
	Disu476	172	172	172	174	172	174	162	172	172	174		
	Disu151	135	135	137	137	135	135	135	135	135	135		
	Disu127	164	164	162	162	162	162	162	162	162	162		
	Disu98	126	126	122	126	98	98	104	104	104	104		
	Disu582	144	144	144	144	144	144	152	152	144	144		
	Disu100	120	122	120	122	120	120	120	120	120	120		
	Disu480	112	112	116	116	116	116	112	116	116	116		
	Disu593	164	166	164	164	166	166	164	164	166	166		
	Disu487	160	160	160	160	160	160	160	160	148	160		
	Disu545	148	148	148	148	148	148	148	148	148	150		
	Disu76	129	129			125	125	125	125			129	129
	Disu269	115	134			152	152	152	152			134	138
	Disu261	150	150			166	166	150	166			150	150
	Disu71	168	172			168	168	170	170			170	172
	Disu33	152	152			164	164	164	164			152	152
	Disu138	161	167			161	167	167	167			155	169

*Individual not tested at all loci due to limited sample availability

Table S3. Tests of 13 microsatellite markers on DNA from Sumatran rhinoceros in Indonesia.

Markers:	Disu033	Disu071	Disu076	Disu100	Disu127	Disu201	Disu261	Disu393	Disu476	Disu593	Disu748	Disu783	Disu863
Amplification success, DNA from blood samples of 3 SRS rhinos	3	3	3	3	3	3	3	3	3	3	3	3	3
Amplification success, DNA from fecal samples of 3 SRS rhinos	2	2	2	3	3	3	3	3	2	3	3	2	2
Blood/fecal genotyping consistency in Andalas	Yes	Yes	Yes	Yes	Yes	Yes	Yes	Yes	Yes	Yes	Yes	Yes	Yes
Amplification success, DNA from 11 fecal samples of wild BBS rhinos	5	10	8	9	11	6	7	9	9	11	9	11	11

For each row, information that identifies the rhinos in each category is listed in Table 1. Thirteen markers were randomly chosen from the 29 available, for the following tests: (1) Genotyping of DNA from blood samples from 3 SRS rhinos. All markers were successful in all three samples. (2) Amplification of fecal samples from 3 SRS rhinos. Two were from individuals from which blood samples had not been collected, for one rhino (Andalas) both types of sample were collected. Two or three of the samples successfully amplified for each marker. (3) For the rhinoceros individual Andalas (Table 1), genotyping results were consistent for DNA extracted from the blood and from the fecal sample, for all lof the markers. (4) Eleven fecal samples were collected in BBS from an unknown number of free-ranging wild rhinos. For each marker, the number of rhinos is listed for which there was successful amplification of DNA extracted from the fecal samples. The 13 markers were chosen at random from 29 available. The marker Disu863 was monomorphic across all samples.

Table S4. Tests of primers on Asian tapir DNA and human DNA

Marker ID	Tapir	Human	Notes
DISU033	NA	NA	
DISU050	NA	NA	
DISU071	183 189	298 298*	
DISU076	NA	285 285*	The peak has no stutter in human
DISU098	NA	NA	
DISU100	NA	NA	
DISU127	NA	NA	
DISU138	NA	NA	
DISU149	NA	NA	
DISU151	?	?	Too much noise (unclear peaks) in both species
DISU201	105 105*	NA	
DISU261	NA	NA	
DISU269	226 226*	?	Too much noise (unclear peaks) in human sample
DISU393	NA	NA	
DISU448	NA	NA	
DISU476	NA	NA	
DISU480	NA	NA	
DISU487	167 167	NA	The peak in tapir has no stutter
DISU501	NA	NA	
DISU542	286 286*	?	Noise (unclear peaks) in human result
DISU545	NA	NA	
DISU556	NA	448 448*	The peak has no stutter in human
DISU582	NA	375 375*	The peak has no stutter in human
DISU593	?	?	Too much noise (unclear peaks) in both species
DISU733	NA	NA	
DISU748	163 163*	NA	
DISU783	NA	NA	
DISU847	NA	NA	
DISU863	159 172 193 194	117 117*	

Shading indicates that human or tapir DNA generated PCR amplicon(s)
NA means no amplification for species; question mark indicates peaks were indistinct ("noise")
Asterisk(*) indicates that the size(s) of the products have a difference >20 bp from the size range in Sumatran rhinoceros

Figure S1 (below). Results for two Sumatran rhino microsatellite makers, each amplified using two methods. The chromatograms are shown for markers *Disu100* (first page) and *Disu593* (second page). DNA samples were amplified for both markers using both AmpliTaq Gold polymerase (top of each panel) and the Qiagen Multiplex PCR Kit (bottom of each panel). The blood sample and fecal sample from SRS were both from Sumatran rhinoceros Andalas (Table 1). The fecal samples from the wild were from free-ranging rhinoceros samples BBS-3-019 (*Disu100*) and BBS-3-005 (*Disu593*). For both markers, the Qiagen Multiplex PCR Kit yielded a better or stronger signal than the AmpliTaq Gold polymerase. The AmpliTaqGold DNA polymerase produced lower quality signals (with no signal in one case using fecal DNA). The scale used may differ across the traces shown.

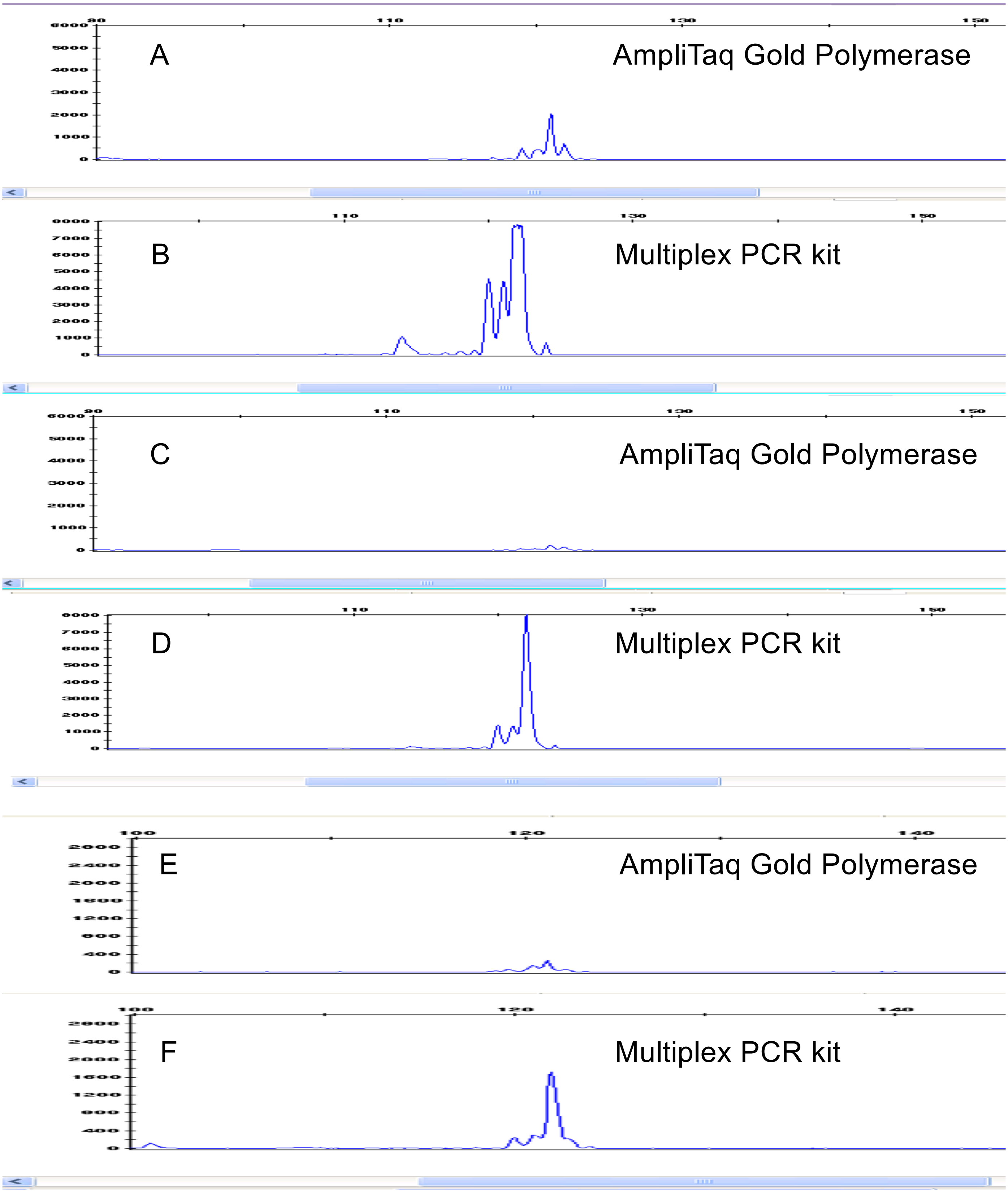

Amplification of *Disu100* fragments using AmpliTaq Gold Polymerase and the Qiagen Multiplex PCR kit

A. Rhinoceros blood sample – AmpliTaq Gold Polymerase
B. Rhinoceros blood sample – Multiplex PCR kit
C. Captive rhinoceros fecal sample – AmpliTaq Gold Polymerase
D. Captive rhinoceros fecal sample – Multiplex PCR kit
E. Wild rhinoceros fecal sample – AmpliTaq Gold Polymerase
F. Wild rhinoceros fecal sample – Multiplex PCR kit

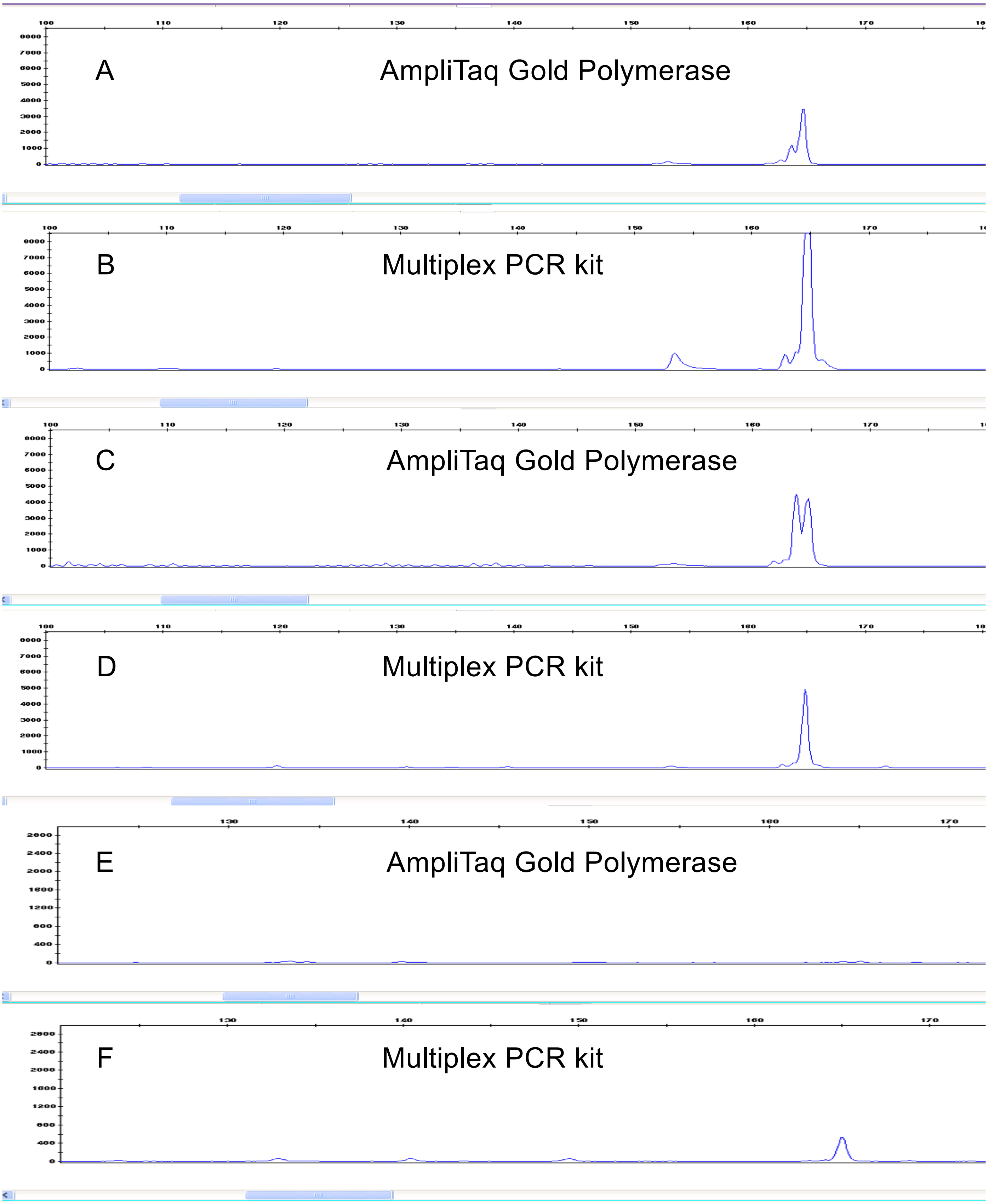

Amplification of *Disu593* fragments using AmpliTaq Gold Polymerase and the Qiagen Multiplex PCR kit

A. Rhinoceros blood sample – AmpliTaq Gold Polymerase
B. Rhinoceros blood sample – Multiplex PCR kit
C. Captive rhinoceros fecal sample – AmpliTaq Gold Polymerase
D. Captive rhinoceros fecal sample – Multiplex PCR kit
E. Wild rhinoceros fecal sample – AmpliTaq Gold Polymerase
F. Wild rhinoceros fecal sample – Multiplex PCR kit

Figure S2. Short tandem repeat motifs and sequences for markers.

Disu033: $(GATA)_{11}$

TCTGGATACCTGAGGCTTGACAATAAATACATGGATACGTACATAGATAGGTAGGTAGG
TAGATAGATAGATTGATAGATAGATAGATAGATAGATAGATAGATAGATAGATAGATAG
GAAGAGGGAAAGAAGTGATGCCAGT

Disu050: $(AT)_{15}$

CTCCCACATTCAGCAAACTTTCAACATTCCTCAGGTAAATTTATATATATACACACACAC
ATATATATATATATATATATATATATATATAAATGAACTGGTTAGGATTATTTCCACGAT
GAAACAGTTTGTAGAGTCATCACTGCCTGG

Disu071: $(TC)_{17}$

TTGAGATGCATTGCCGTGGGGAATCCAGAAGTCTCTCTCTCTCTCTCTCTCTCTCTCTC
TCTCCCTCTCTCTCATATCTGAACATGCGTCTCATTCTTCTCTCCCTGTAGACTGATTTCTT
CTGTTCCTCATCCACGATGCAGAAACCATGG

Disu076: $(AC)_{13}G(AC)_{7}$

TTCCAGCCGCTCTTATGACCTACACACACACACACACACACACACACGCACACACACAC
ACATTCAAGAGAACATTCTCTCTTCAAAGAAGATAAACAGATGGCCAATAAGCACATGA

Disu098: $(GT)_{9}$

GCTAGGAGAGGGTGTTGGACTGCGTGCGTGCNTGCGTGCGTGCGTGCGTGTGCGTGCGC
GTGCGCGTGTGCGTGTGTGTGTGTGTGTGTTAGGAAAGAGGCAAGGCTACCA

Disu100: $(TA)_{10}$

TGTGGACTTGTCATATATGGGCTTTATATATATATATATATATAATGGACTATTATTCAGC
TATAAAAAAGAAAAAAATCCTGTCATTTGTGACAGCATGGATGAA

Disu127: $(AC)_{10}$…$(AC)_{11}$…$(AC)_{6}$

CCACCACCACCATGCATAGACACACACACACACACACACAGACACACACAGACACACAC
ACACACACACACACAGACACACACACACATACAGTTGATCTCTGCTTCATTGGAAGGGC
CTTCTCATGGCTTCAGCATGGGAGCAAATG

Disu138: $(TG)_{8}$

GGGACACATGACTCCTCTTATCAGATATGTGTATTTTGTTTAAAAAGGAACATTTCACTA
AATGTTTCCAGACAGAATTTTTTTAAGTATATATATATGTGTGTGTGTGTGTGTATGTGGT
AGTATAAGGTGGAGTGG

Disu149: $(AG)_{11}AA(AG)_8$

GAGCGTGCATGGTAGTTTCCAAAGAATATACATTATAGGTATATATACATAGAGAGAGA
GAGAGAGAGAGAGAAAGAGAGAGAGAGAGAGATACTGATGGGAATTAGTCGCACATGC
AAATCTACTCCGTCTGCTATGAGAACC

Disu151: $(TG)_9$…$(TA)_7$

CATTGTGCTCGCTACGCAGAGGCTTTGTGTGTGTGTGTGTGTGTAGATAGATAGATACTA
TATATATATATACATAGAATGTGTGCGTATGTACATAGTCCCCTGGCTCTTGACACCTAG

Disu201: $(AC)_{10}G(CA)_7$

TGGAGAGAATTTCAGACATGGGAGAATATTTCAGTACATTCCCCAACACACACACACAC
ACACACGCACACACACACACAGAGTTTGGACTGAGTTTCATTCCTAGTTTTGCTATTTAT
CTGCCAATGGATCTTGGGCTAG

Disu261: $(CA)_{13}$

AAACCATACGCGGGAGAAGGCTGGGAAACATTTTTAAGCTACTCGTGCACACATACACA
CACGCGCGCGCACACACACACACACACACACACACCACACTTTGAAATAAGCAAG
CACACTGGGGCTCCTGCATGATCTTCCCTTC

Disu269: $(TG)_{12}$…$(TG)_{15}$

CAAGACCACACCTGCTTGTCTTTGTGTGTGTGTGTGTGTGTGTGTGTCTTTGTGTGTGTCT
TTGTGTGTGTGTGTGTGTGTGTGTGTGTGTGTAGCGGGCTGGGTGATGAGTGAGT

Disu393: $(GA)_7$

AGTGAGCAAGGGAATGTGTGTGTGGGGACAGTGAGTGTGCAACAGTGTGAGAGAATATG
TGTGAATGTGTGACCATGTGTGTGTGTGAGAGAGAGAGAGATGTGTATCAGTCTGGGTCC
AATCAAGAGACAGCACCC

Disu448: $(TG)_{10}$

CAGGTTTCGTTACTGCAGGACTTTTCAGAGCCTTTAATCTACAAATTGTGTGTGTGTGTGT
GTGTGTATGCATGTGTCTTGGTTTCCCAAACAACTCTGACTGCAGAACCCTTTGGCTTGTG
CATCTCAGGTCACCAGA

Disu476: $(TC)_6$…$(TC)_9$…$(CA)_{10}$

AAACAGGGAAACAAGGTGCGTCTGTCCTTCTTTCTCTCTCTCTCCTCTCTCTCCATCTCTC
CATCTCTCTCTCTCTCTCTCCCTCCCACACACACACACACACACAGTGCTTTTTTAAAATC
AACCCTAACAGAAAGGGCGCAGTC

Disu480: $(TG)_{11}(GC)_7$

CCTGCCTTCTAGTCCTGTGGAAACTGTGTGTGTGTGTGTGTGTGTGCGCGCGCGCGCGCG
CGGTCCTGTGTAAATCACTTAGCCTTCCTGATCCTGCTTGCT

Disu487: $(CA)_6$…$(CA)_7$

TATCATGTCACAAGCACGCGTGCATGAATGTGCACGTGTGCACACACACACACGCGCAC
ACACACACACACTTACCCCCACAGAGATCTGGGGAGCTACCATTGGAACCTGAGGTGGG
GTGTTGGTGCTGTCGTGAAGAAGAC

Disu501: $(TA)_6$…$(TA)_6$…$(TA)_6$

TGGCCACATCTTCAGCATTAAGATTATAGTTAATATTGTGTGTATATATATATATGTATAT
ATATATATGTGTATATATATATACATATATATTAGTTATGTGACTGCTGTGTGGGGCCTGT
AACTGTGTTAGGTGC

Disu542: $(CA)_8CT(AC)_7$

AAACTACAGGCACGTACAGCCACACACACACACACACTACACACACACACACCACACTC
CCTTCGTCACTCCACACTTTGTGCCTGTGCTCATTTCAGACCGCACCTCATCTCTCAA

Disu545: $(GA)_8$…$(AG)_6$

TGTTGTCCAAGCTGTGTCTGTGTGCGTGTGTGTGTGTATGTGTGTATGTGAGAGAGAGAG
AGAGATTGTGATAGAGAGAGAGAGAAGTATATTCATTAACCCATCTATTTATTCCTGTTA
GGTACCAGCTGCCA

Disu556: $(GT)_7$…$(AT)_6$

GCCAATTAAATCTACCTGCCACTGAAAAAAGCATATATATGCGTGTGTGTGTGTGTACTT
TAATATATATATATTTAATATATGTTTTAATATATCTATATCTATTTCTATATCTCAATATT
GTTATAATACCTGGGTTTGAGTCTTGGC

Disu582: $(AC)_{10}$…$(AC)_{11}$

TCTGTGGTGGTAGCTGTGACAACACACACACACACACACACATACACACACGCACGCAC
ACATACACACACACACACACACACACCCATCCATACATCCACATCCATGTGTACATGGGT
GTCTCTGTGCCA

Disu593: $(AC)_6$

CCACGTCCCAGGTCAAGAGGTGTGTGTGTATATTTAAATCACCTGTAGTCTCTACACACA
CACACCCACACATCCATAGCCTTTATTATCTCTGTATGGTTAGGGATCATTTTCTCTTATT
TCTAAACTGAGCCACCAGGAACAGCT

Disu733: $(GT)_{11}$…$(TG)_{10}$

TGGCACAGAGACACCCATGTACACATGGATGTGGATGTATGGATGGGTGTGTGTGTGTGT
GTGTGTGTATGTGTGCGTGCGTGTGTGTATGTGTGTGTGTGTGTGTGTTGTCACAGCTA
CCACCACAGA

Disu748: $(TC)_9C(CT)_{12}$

CCTTGATTGGTGGGTTCCCTTTCTCTCTCTCTCTCTCCCTCTCTCTCTCTCTCTCTCTC
TTTCACGTGCAGGCACACACACGTGCGCTCTCTCT

Disu783: $(GT)_6C(TG)_6C(GT)_9$

CCTTGCCTTGCCTTCAATCCTCAAAAGTCTGCTACACCACAAAGAACGTGTGTGTGTGTC
TGTGTGTGTGTGCGTGTGTGTGTGTGTGTCTGTGTAGGAGAAAGGATGG

Disu847: $(CA)_{10}…(CA)_{10}$

AAAGTCGCCTCTCACACACCCAGGCCAGCCCGCAGGGCCTGCCCTCCCAGCACACACAC
ACACACACACACACTCTCACACACACACACACACACACTCACTCGGCCGCTTACAAG
GAGGCTCTGA

Disu863: $(CA)_6$

GAAGCTGTATGTCCGGATGCACACACACACACGCACGCACGCACGCACATATATTTGAT
ATACATACACAGGTATATATATACATCAAATATATAGAATGCATGTAATGGCAATGAGA
TGCAGTTACTCTGAGGAAGGTCTGTTTAGC
